# Whole-brain 3D mapping of human neural transplant innervation

**DOI:** 10.1038/ncomms14162

**Published:** 2017-01-19

**Authors:** Jonas Doerr, Martin Karl Schwarz, Dirk Wiedermann, Anke Leinhaas, Alina Jakobs, Florian Schloen, Inna Schwarz, Michael Diedenhofen, Nils Christian Braun, Philipp Koch, Daniel A. Peterson, Ulrich Kubitscheck, Mathias Hoehn, Oliver Brüstle

**Affiliations:** 1 Institute of Reconstructive Neurobiology, University of Bonn, Sigmund-Freud-Strasse 25, 53127 Bonn, Germany; 2 Life&Brain GmbH, Sigmund-Freud-Strasse 25, 53127 Bonn, Germany; 3 Department of Epileptology, Functional Neuroconnectomics Group, University of Bonn, Sigmund-Freud-Strasse 25, 53127 Bonn, Germany; 4 Max Planck Institute for Metabolism Research, In-vivo-NMR Laboratory, Gleuelerstrasse 50, 50931 Cologne, Germany; 5 Institute of Physical and Theoretical Chemistry, University of Bonn, Wegeler Strasse 12, 53115 Bonn, Germany; 6 Center for Stem Cell and Regenerative Medicine, Rosalind Franklin University of Medicine and Science, 3333 Green Bay Road, 60064 North Chicago, Illinois, USA

## Abstract

While transplantation represents a key tool for assessing *in vivo* functionality of neural stem cells and their suitability for neural repair, little is known about the integration of grafted neurons into the host brain circuitry. Rabies virus-based retrograde tracing has developed into a powerful approach for visualizing synaptically connected neurons. Here, we combine this technique with light sheet fluorescence microscopy (LSFM) to visualize transplanted cells and connected host neurons in whole-mouse brain preparations. Combined with co-registration of high-precision three-dimensional magnetic resonance imaging (3D MRI) reference data sets, this approach enables precise anatomical allocation of the host input neurons. Our data show that the same neural donor cell population grafted into different brain regions receives highly orthotopic input. These findings indicate that transplant connectivity is largely dictated by the circuitry of the target region and depict rabies-based transsynaptic tracing and LSFM as efficient tools for comprehensive assessment of host–donor cell innervation.

## Abstract (web summary)

Transplantation of cells into the central nervous system has developed into a major avenue for replacing neurons lost to neurodegenerative disease. Here the authors develop an approach combining viral-based transynaptic tracing labeling and whole brain imaging to trace synaptic innervation of human neurons transplanted into a mouse background.

Transplantation of cells into the central nervous system has developed into a major avenue for replacing neurons lost to neurodegenerative disease^1^,^2^. The suitability of neural transplants for restoring or modulating neuronal function critically depends on the ability of donor cells to engage in synaptic interaction with the host brain circuitry, and appropriate model systems are required to assess this integration process in a preclinical scenario. Conventional electrophysiological methods such as patch clamp analysis are limited in that they require tedious identification of ‘pairs' of connected cells, which restricts their applicability to small numbers of neurons per brain^3^. While optical methods such as calcium imaging or voltage-sensitive reporters can extend resolution to entire neuronal ensembles, they require close proximity between the recording device and the recorded cells, for example, via prior sectioning of the tissue or direct access to localized brain regions through cranial windows^4^,^5^. However, a comprehensive assessment of transplant integration should ideally enable coverage of all transplanted cells and host connection partners throughout the recipient brain.

Rabies virus (RABV)-based systems exploit the property of this virus to undergo retrograde transsynaptic transport^6^. Genetically modified RABV variants carrying fluorescence reporter genes have been used successfully to visualize synaptically connected neurons^7^. Recently, optical tissue clearing methods in combination with light sheet microscopy have emerged as highly useful techniques for microscopic analysis of tissue fragments and even entire organs without the need for mechanical sectioning^8^,^9^,^10^,^11^,^12^,^13^,^14^,^15^.

Here we set out to design an approach combining RABV-based transsynaptic tracing, tissue clearing, light sheet fluorescence microscopy (LSFM) and magnetic resonance imaging (MRI) co-registration to enable qualitative and quantitative assessment of human transplant innervation in the context of an entire mouse brain.

## Results

### Transsynaptic tracing of graft innervation

**Figure 1:**
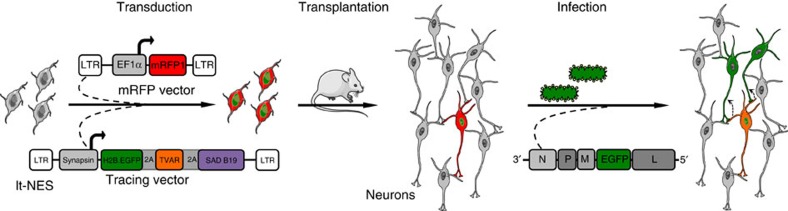
Experimental procedure for tracing host-graft connectivity. Lt-NES cells were transduced with two lentiviral vectors expressing mRFP1, and an H2B.EGFP fusion protein coupled via 2A peptides to the TVA receptor and the RABV B19 glycoprotein. Ten weeks after transplantation the grafts were infected with RABVΔG-EGFP(EnvA), which enables monosynaptic retrograde tracing of afferent host neurons. Parts of the schematic were produced using Servier Medical Art (http://www.servier.com).

To trace synaptic innervation of human neurons in a mouse background, we employed long-term self-renewing neuroepithelial stem cells (lt-NES cells) derived from human embryonic stem cells. Lt-NES cells represent a stable neural stem cell population, which can be extensively propagated while maintaining a stable neurogenic potential yielding fully functional neurons both *in vitro* and following transplantation into the rodent brain^16^,^17^. Due to their robust proliferation and differentiation potential, lt-NES cells lend themselves particularly well to genetic modification and have been successfully used for lineage tracing, disease modelling and cell-mediated gene transfer^18^,^19^,^20^. They exhibit a posterior phenotype with an anterior hindbrain identity, and mostly give rise to GABAergic interneurons as well as glutamatergic neurons, a differentiation pattern maintained after transplantation into neonatal and adult hosts^16^,^21^. For the present study we generated lt-NES cells ubiquitously expressing mRFP1 to label all transplanted cells, and a synapsin promoter-driven combination of the avian TVA receptor, the B19 rabies glycoprotein and a H2B.EGFP (enhanced green fluorescent protein) fusion protein. The latter construct enables infection of the transfected cells by pseudotyped, glycoprotein-deleted RABV, replication of the virus and tracing of infected cells as well as first order synaptically connected neurons (Fig. 1)^22^. Lt-NES cells were stereotaxically delivered to the striatum or the hippocampal dentate gyrus of adult unlesioned immunodeficient *Rag2*^−/−^ mice. The immunodeficient host background was chosen in order to enable long-term survival of the transplanted xenogeneic cells^23^. Ten weeks later the human graft (Supplementary Fig. 1a) was infected by stereotaxic injection of recombinant pseudotyped, glycoprotein-deleted EGFP-expressing RABV (RABVΔG-EGFP(EnvA))^6^. Mice were killed 10 days following injection of the virus. This 10-day time span is sufficient for replication of the virus in the grafted human cells and its retrograde propagation to afferent neurons^24^. To enable whole-mount visualization, fixed entire brains were cleared in a benzyl alcohol/benzyl benzoate-based solution (FluoClearBABB) that enables long-term preservation of fluorescent proteins^15^ and subsequently subjected to LSFM.

### 3D imaging of neural grafts and afferent host neurons

**Figure 2:**
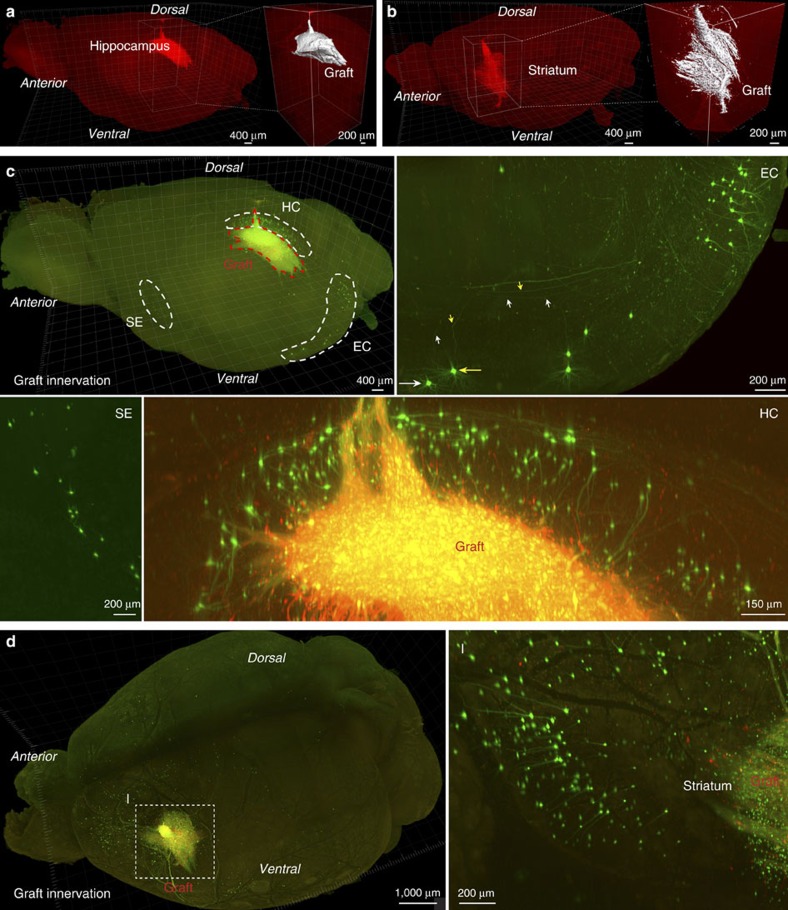
Whole-mount imaging of grafted human lt-NES cells. (**a**,**b**) Human grafts in the hippocampus (**a**) and striatum (**b**) as detected by their mRFP1 expression. Insets depict magnified graft cores as determined by 3D surface rendering. (**c**,**d**) Co-visualization of mRFP1 and EGFP fluorescence identifies RABVΔG-EGFP-infected donor cells while exclusively EGFP^+^ cells represent retrogradely labelled host neurons connected to engrafted neurons. (**c**) Hippocampal grafts (HC) receive orthotopic input from the entorhinal cortex (EC; coloured arrows indicate neurons and their corresponding axon), the septum (SE) and intrahippocampal host neurons within the pyramidal cell layer and stratum oriens. Striatal grafts (**d**) are largely innervated by cortical neurons as well as adjacent striatal neurons. Shown are representative images from *n*=3 cleared brains per transplantation site; scale bars as indicated.

**Figure 3:**
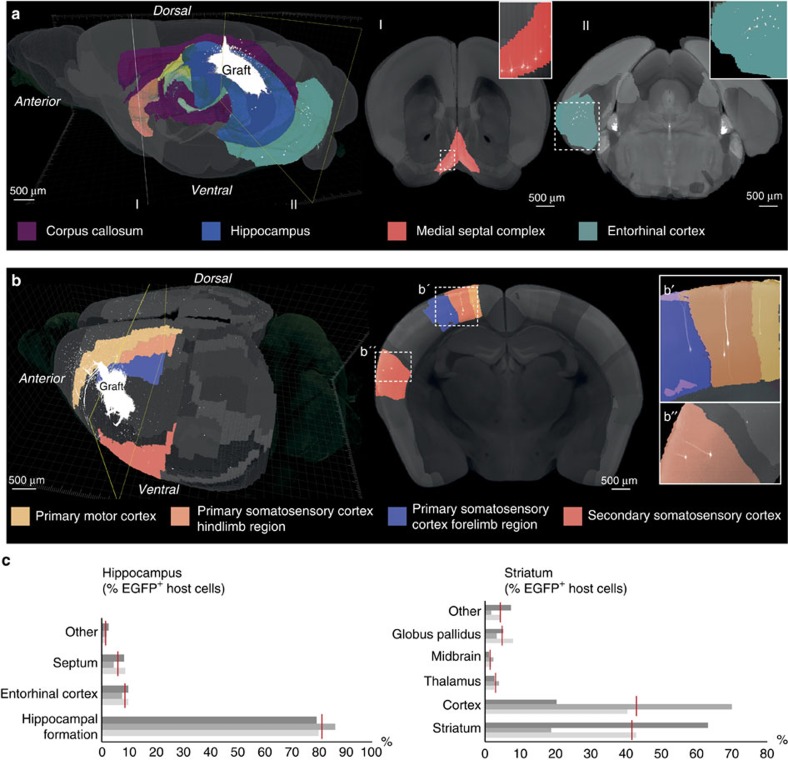
Anatomical distribution of host neurons projecting onto grafted human neurons. Simultaneous representation of LSFM-recorded EGFP-labelled neurons and co-registration with MRI data in recipient mouse brains with (**a**) hippocampal and (**b**) striatal grafts. Host cells innervating the hippocampal graft are allocated to adjacent hippocampal territories, septum and entorhinal cortex. In brains harbouring striatal grafts, MRI co-registration permitted the allocation of host input neurons to cortical subregions such as motor cortex, secondary somatosensory cortex and distinct areas within primary somatosensory cortex. Shown are representative images from *n*=3 cleared brains per transplantation site; scale bars as indicated. (**c**) Quantification and anatomical allocation of EGFP^+^ host neurons innervating the grafted human neurons. Red vertical bars indicate mean values for 3 recipient animals each (grey horizontal bars). Shown is the percentage of all detected EGFP^+^ host neurons.

Human grafts labelled with mRFP1 were clearly discernible in the two target sites (Fig. 2a,b). EGFP fluorescence revealed a large fraction of double labelled cells reflecting RABVΔG-EGFP(EnvA)-infected donor neurons (Fig. 2c). In addition, numerous EGFP-only-positive cells were detected in the vicinity of the grafts as well as in more remote regions of the recipient brains, indicating retrograde labelling of synaptically connected host neurons projecting onto the transplant-derived neurons (Fig. 2c,d). Interestingly, analysis of traced neurons revealed localization of the cells predominantly in orthotopic projection areas. Mice with hippocampal grafts showed numerous EGFP^+^ cells in the entorhinal cortex and the septum (Fig. 2c), that is, two predominant input regions of the hippocampus. EGFP-only-positive neurons were also found inside the hippocampal formation, in particular within the pyramidal cell layer of the CA1 region and the stratum oriens, indicating innervation of the graft by resident hippocampal neurons (Fig. 2c). In contrast, mice with striatal grafts contained numerous EGFP-only-positive cells with projection neuron morphologies in the cortex, a major input area of the striatum (Fig. 2d; Supplementary Fig. 1b)^25^. Digital reconstruction of the LSFM data sets enabled a detailed representation of the transplanted neurons and their entire afferent connectome in a single specimen with sufficient morphological resolution to detect neurites and archetypical morphologies of host neurons in the different input regions (Supplementary Movies 1 and 2; Supplementary Fig. 1b). The innervation patterns of hippocampal and striatal grafts were virtually indistinguishable from native afferents of these regions as visualized in a parallel experiment by direct stereotaxic delivery of a tracing virus cocktail (rAAV (mRFP1/TVA receptor), rAAV (B19 glycoprotein) and RABVΔG-EGFP(EnvA)) into the hippocampus and striatum of untransplanted hosts (Supplementary Fig. 1c,d)^22^,^26^. Similar innervation patterns were obtained upon transplantation of an independent human induced pluripotent stem cell-derived neural precursor line generated with a different protocol (small molecule-induced neural precursor cells, Supplementary Fig. 2)^27^. Only very occasional EGFP^+^ host neurons were detected outside the neuroanatomically well-documented major input regions of the two transplant target regions (Fig. 3c; Supplementary Movies 1 and 2; see also Supplementary Table 1). Taken together, these data indicate that lt-NES cell grafts in the dentate gyrus and the striatum receive afferent innervation highly similar to that of endogenous hippocampal and striatal neurons.

### Anatomical allocation of input neurons via MRI co-registration

A major disadvantage of tissue clearing techniques is that anatomical landmarks are no longer visible within the translucent tissue samples, which severely impedes the precise spatial allocation of fluorescently labelled cells to distinct host brain compartments. We have addressed this problem by co-registering our LSFM data sets with high-precision three-dimensional (3D) MRI reference data sets (for details see ‘Methods' section)^28^,^29^. This approach employs established algorithms to extrapolate spatial anatomical relationships from averaged reference brain samples to the individual specimen. Co-registration data confirmed orthotopic innervation of human lt-NES cell grafts within the dentate gyrus by host neurons in the adjacent stratum oriens and CA1 regions as well as from neurons within the ipsilateral entorhinal cortex and the medial septal complex (Fig. 3a). For recipient brains with striatal grafts co-registration enabled a much more precise allocation of afferently connected host neurons, which were not only detected in cortex and the adjacent striatal tissue, but also in thalamus and the globus pallidus. Furthermore, EGFP^+^ host neurons projecting onto the graft could be allocated to cortical subregions such as distinct areas within motor cortex and somatosensory cortex (Fig. 3b). The depicted innervation patterns correspond to endogenous trajectories reported in connectivity analyses for endogenous neurons^26^,^30^ and underline the remarkable degree of orthotopic innervation of grafted lt-NES cells.

We then used the acquired 3D data to explore whether whole-mount LSFM and co-registration with MRI data sets could, in principle, permit a quantitative assessment of afferently connected host neurons in a single whole-brain preparation by allocating EGFP^+^ host cells to anatomical compartments. Employing co-visualization of MRI-derived anatomical information and LSFM data we could stratify the number of connected host neurons across the major input regions. In animals that received hippocampal grafts, the main afferents originated from the hippocampal formation itself, the entorhinal cortex and the septum. For grafts in the striatum, EGFP^+^ afferent neurons could be observed in the cortex, the globus pallidus, the thalamus and the striatum itself (Fig. 3c; Supplementary Table 1). Despite the observed variations, which might reflect inherent variability in surgical delivery and distribution of the transplanted cells, these data indicate that LSFM and co-registration with MRI reference data can largely facilitate quantitative assessment of connectivity analyses and other applications such as migration studies.

## Discussion

Our data demonstrate that RABV-based transsynaptic tracing in combination with LSFM of cleared brain samples and co-registration with MRI reference data enables qualitative and quantitative assessment of the afferent connectome of transplanted human neurons in a single whole-mount specimen with high anatomical and morphological resolution. From a biological point of view, the high degree of orthotopic innervation of lt-NES cell grafts in both hippocampus and striatum is remarkable, in particular since lt-NES cells are known to exhibit a posterior phenotype corresponding to an anterior hindbrain location^16^. Our observations are reminiscent to an earlier study, which demonstrated that lt-NES cells grafted into the adult unlesioned hippocampus or cortex develop region-specific efferent projections onto orthotopic host brain targets^21^. Collectively, the results of these studies suggest that establishment of afferent and efferent connections between host brain and grafted human neural stem cells is largely dictated by the topology of pre-existing, endogenous fibre tracts and circuitries rather than the regional identity of the donor cells.

We expect the experimental system presented here to serve as a useful tool for a number of biomedical applications. Striatal transplantation of dopaminergic and GABAergic neurons is intensely pursued as therapeutic strategy for the treatment of Parkinson's and Huntington's disease, respectively^31^,^32^,^33^, and the approach presented here could enable efficient assessment of different donor cell populations with respect to their ability to integrate synaptically into the host brain circuitry. We also anticipate that our system will become useful for studying the phenotype of patient-derived neurons *in vivo*. Since their first description by Shinya Yamanaka^34^,^35^, iPS cells have become a major tool for modelling human diseases *in vitro*. However, *in vitro* studies are limited with respect to follow-up time and questions relating to cell-tissue interactions. The approach reported here could tackle some of these challenges and enable the assessment of synaptic integration of patient-specific iPS cell-derived neurons in an *in vivo* setting, thereby facilitating mechanistic studies into the pathogenesis of neurodevelopmental, neuropsychiatric and neurodegenerative disorders. As for the latter, transsynaptic spread of disease has become a major topic of research^36^,^37^,^38^, and visualization of synaptic connections onto grafted patient-derived neurons may provide a formidable tool to mechanistically dissect this phenomenon in an experimental *in vivo* scenario. Finally, our system might also be helpful for benchmarking the synaptic integration potential of novel synthetic cell sources such as neural precursors and neurons generated by direct transcription factor-based cell fate conversion^39^,^40^,^41^,^42^.

## Methods

### Neural stem cell culture

Long-term self-renewing neuroepithelial like stem cells (lt-NES cells; derived from embryonic stem cell line I3) were generated by manual isolation of rosette-like structures of plated neuralized embryoid bodies as described previously^16^ and continuously propagated on poly-L-ornithine/laminin-coated plates in N2 medium supplemented with B27 (1 μl ml^−1^, Invitrogen), 10 ng ml^−1^ FGF2 and 10 ng ml^−1^ EGF (both from R&D systems). Neuronal differentiation was induced by removing the growth factors FGF2 and EGF from the media and culturing the cells in Neurobasal medium supplemented with B27 (1:50, Invitrogen) and DMEM/F12 supplemented with N2 (1:100) mixed at a 1:1 ratio and supplemented with 300 ng ml^−1^ cAMP for ≥4 weeks. Cells were routinely tested for mycoplasma contamination.

### Vector design and lentiviral transduction

In order to introduce labelling and tracing constructs into lt-NES cells, we employed two distinct lentiviral vectors. For permanent labelling of the cells, a construct constitutively expressing mRFP1 was used (pLentiWE-Ef1α-mRFP1; Life Technologies). In order to restrict expression of the tracing cassette to mature human neurons we used a synapsin promoter driving the coturnix japonica Rous sarcoma virus receptor (TVA-R) recognizing the subgroup A avian sarcoma and leukosis virus (ASLV-A)-pseudotyped, glycoprotein-deleted RABV, Histon2B.EGFP nuclear fusion protein as well as the rabies SAD B19 glycoprotein all linked by a self-cleaving 2 A peptide sequence (2A; porcine Teschovirus-1, taken from pBOB-synP-HTB, Addgene plasmid #3019). To facilitate fast enrichment of lentivirally transduced cells, the expression cassette was transferred to the pLVXTP backbone which contains a puromycin resistance gene (Clonetech) by opening it using *XhoI* and *MluI* digestion and ligation with the PCR amplified (forward primer 5′-GCAGCAGTCGACACTGCAGAGGGCCCTGC-3′; reverse primer 5′-GCAGCAACGCGTTTGTCTGAGGTGTGACTGGAAAACC-3′) and *SalI*/*MluI* digested tracing cassette. Lentiviral particles were generated and concentrated using the polyethylene glycol method as described previously^20^,^43^. Briefly, 293FT cells (Life Technologies) were transfected with the lentiviral plasmids pLVX-SynHTB or pLentiWE-Ef1α-mRFP1 and the packaging plasmid psPAX2 and the envelope plasmid pMD2.G. Supernatant was collected on day 2 and day 3 after transfection, filtered (0.45 μm) and enriched by centrifugation. Lt-NES cells were transduced with enriched pLentiWE-Ef1α-mRFP1 supernatant. Two days later they were subjected to a 3-day selection with 20 μg ml^−1^ blasticidin (Life Technologies). Eleven days later, cells were transduced with concentrated pLVX-SynHTB supernatant. Two days later they were subjected to a 3-day selection with 1 μg ml^−1^ puromycin (Sigma-Aldrich).

### Production of pseudotyped rabies virus

BHK cells (∼1.5 × 10^7^; ATCC CCL-10) were plated in a 15 cm Petri dish. The following day, cells were transfected with 15 μg plasmid DNA (pCAGG/SAD-G) by calcium phosphate transfection. Twenty-four hours later RABVΔG-EGFP was added at a multiplicity of infection (MOI) of 3. After additional 48 h incubation the RABVΔG-EGFP containing supernatant was equally distributed onto four 15 cm plates containing pCAGGs/SAD-G (15 μg per plate) transfected BHK cells (∼1.5 × 10^7^ cells per plate)^6^. Two days later the virus-containing supernatant was applied onto four 15 cm plates containing BHK-EnvARGCD cells (∼1.5 × 10^7^ cells per plate) at a MOI of 1.5 for pseudotyping. After 12 h cells were trypsinized and replated onto eight dishes. Pseudotyped RABV (RABVΔG-EGFP(EnvA))-containing supernatant was collected 2 days later. The supernatant was cleared by 10 min centrifugation at 2,000 r.p.m. at 4 °C and subsequently filtered through a 0.45 μm filter (Nalgene SFCA Bottletop Filter, ThermoFisher Scientific). The virus was pelleted by 90 min centrifugation at 25,000 r.p.m. (SW28, 4 °C) in a Beckman 80 K ultracentrifuge (Beckman Coulter). After centrifugation the supernatant was discarded. The virus pellet was aspirated in ice-cold PBS (pH 6.8) and stored at −70 °C. Virus titres were determined by serial dilution and overnight infection of primary cortical neurons that had been infected with rAAV1/2 expressing TVA IRES mCherry under control of the human synapsin promoter. Three days later the number of fluorescent SADΔG-EGFP containing neurons were counted. Titres of RABVΔG-EGFP(EnvA) used for *in vivo* injections were ∼2.5 × 10^7^ ml^−1^.

### Transplantation of *Rag2* ^−/−^ mice

Cell transplantation into the hippocampus (*n*=21) or striatum (*n*=17) of adult female mice (8 weeks; *Rag2*^−/−^ (genetic background: B10;B6-Rag^2tm1Fwa^ Il2rg^tm1Wjl^), Taconic Biosciences Inc.)^23^ was performed as described previously^21^. Briefly, lt-NES cells were trypsinized, treated with DNAse (0.1% at RT for 10 min), filtered through a cell mesh, spun and re-suspended in Cytocon Buffer II (∼60,000 cells μl^−1^ or 40,000 cells μl^−1^, as indicated). Analgesia and anaesthesia of mice was initiated using a mixture of carprofen (5 mg kg^−1^ BW i.p.), fentanyl (0.05 mg kg^−1^ BW i.p.), midazolam (5 mg kg^−1^ BW i.p.) and medetomidine (0.5 mg kg^−1^ BW i.p.). Heads were shaved and sterilized and positioned in the stereotaxic frame. The scalp was opened sufficiently to reveal lambda and bregma points. A stereotaxic apparatus (Stoelting) was used to target the desired coordinates (2.4 mm posterior, 1.5 mm lateral and 1.4 mm ventral for hippocampal grafts and 0.8 mm anterior, 1.8 mm lateral and 2.7 mm ventral for striatal transplants). To penetrate the skull, fascia and periosteum were removed with forceps, and a burr hole was drilled using a bone drill while retaining a clean and sterile area. The dura was perforated using a surgical scalpel, and a glass injection needle containing 1 μl cell suspension was lowered slowly (within 30 s) down to the target depth. The cell suspension was injected carefully (over 3–15 min dependent on resistance), the needle was withdrawn slowly, and the hole in the skull was sealed by application of bone wax. The skin was closed with wound clips and the anaesthetic was antagonized by injection of naloxone (0.4 mg kg^−1^ BW i.p.), flumazenil (0.5 mg kg^−1^ BW i.p.) and atipamezole (5 mg kg^−1^ BW i.p.). Mice were transferred to a warming pad for standard post-operative care. Ten weeks later, RABVΔG-EGFP(EnvA) was used to infect the grafted cells via the same stereotaxic approach. Mice were killed 10 days later by perfusion fixation. From the 38 transplant recipients, 32 were used for conventional assessment of cell survival, cell differentiation and migration and to determine the optimal time point and follow-up window for RABV infection of the graft. For each transplantation site 3 randomly selected mouse brains were subjected to the clearing procedure, subsequently imaged by LSFM and included into the co-registration process. The studies were conducted in accordance with the legal requirements of the local authorities (permit number: AZ84.02.04.2013.A368).

### Immunohistochemical analysis

Ten days following injection of RABVΔG-EGFP(EnvA), mice were anaesthetized with ketamine (80 mg kg^−1^; Pfizer)/xylazine (10 mg kg^−1^; Bayer), and subjected to perfusion with PBS/Heparin (1,000 I.E. ml^−1^, Ratiopharm) followed by PFA (4%, 4 °C, Sigma). Coronal sections (30 μm) were generated using a cryostat, dried, reconstituted and washed in PBS for 30 min at room temperature.

The expression of human nuclei marker was analysed by blocking the sections in 10% normal horse serum (NHS), 1% normal goat serum (NGS) and 0.1% Triton X-100 in PBS and applying primary antibody (hNuc; Millipore, MAB4383; 1:500, o/n) and secondary antibody (Alexa 555, A-21422; Thermofisher; 30 min) in 3% NHS, 1% NGS and 0.1% Triton X-100 in PBS.

### Tissue clearing and light sheet fluorescence imaging

For clearing of whole adult mouse brains, specimens were treated as previously described^15^. Briefly, tissue was dehydrated using a tert-butanol-based ascending alcohol series at 30 °C (pH 9.5) and mounted in a triethylamine pH-adjusted benzyl alcohol/benzyl benzoate mixture (BABB, pH 9.5). A purpose-built light sheet fluorescence microscope was used to visualize the samples at a voxel size of approximately 1.6 × 1.6 × 3.2 μm^3^, resulting in ∼60 GB of stitched 16 bit raw data sets per channel and hemisphere^15^.

### 3D reconstruction of imaged data

For generation of traverse Z-projections, the maximum intensity projection algorithm of Fiji was used (http://pacific.mpi-cbg.de/wiki/index.php/Fiji). The representation of the generated data as a 3D object was performed using the Surpass view in Imaris while virtual sections of varying thickness were generated using the extended feature of the slice tool (Bitplane). Data processing was performed on a custom-built power workstation equipped with two Xeon E5-2667v3 CPUs, 512 GB memory and a Nvidia Titan-X GPU.

### Co-registration

Briefly, mapping anatomical regions to the stack of LSFM images was done by co-registering downsized microscopy images to a MRI template, which was connected to dedicated mouse brain atlases. Applying the inverse of the computed transformation matrices to the atlases aligns them to the individual microscopy images.

Coronal MRI images (RARE, TE/TEeff=10.8/32.5 ms TR=5,500 ms, RARE-factor=8, NA=2) of 9-week-old, healthy C57 Bl6 mice (*n*=24) with an in-plane resolution of 68 and 200 μm slice thickness were acquired on a 9.4T Bruker Biospec 94/20, using a Bruker Cryoprobe. The individual MR-images were co-registered and those co-registered images were averaged to form a template covering the normal anatomical variations of the mouse brain as a reference data set for further processing.

Two MRI atlases referencing different anatomical structures on MRI images in the mouse brain were combined, and their labels were merged^28^,^29^. The MRI template was co-registered once to the atlas resulting in a transformation matrix M_1_.

LSFM images were cropped to an in-plane resolution of a multiple of 10 (for example, 7,350 × 5,100 pixels) and then downsized in-plane by factor of five gaining a set of images of medium resolution (for example, 1,470 × 1,020 pixels) to keep computational time for further processing reasonable. As the clearing procedure results in tissue shrinkage, the microscopy data were scaled during the co-registration process to fit the template (scaling factors ranged between 1.28 and 1.40; average 1.37±0.09). The data set was flipped from axial to coronal orientation to match the orientation of the MRI template and atlas. The green fluorescence channel of each subject was further sampled down by an additional factor of 2 in plane and a factor of 4 for the number of slices, gaining a data set with a resolution more similar to the MRI atlases. The background was masked to zero intensity before it was co-registered using flirt Ver. 5.5 from FSL Ver. 4.1.9 (refs 44, 45), to the MRI template resulting in a transformation matrix M_2_. The combination of M_1_ and M_2_: M_1_M_2_=M_3_ or its inverse M_3_^−1^ was then used to transform and overlay either the medium sized LSFM images to the atlas or the atlas to the LSFM data, respectively.

### Stereology

Quantitation of the number of transplanted mRFP1-positive human cells was performed based on 3D light sheet microscope data sets using the Optical Fractionator approach^46^,^47^ with StereoInvestigator software (MBF Bioscience, Inc., Williston, VT). The fractionated sampling scheme has the advantage of being insensitive to the volumetric distortion produced by clearing^47^. Systematic random sampling of tissue was performed in virtual horizontal sections set at a defined section height of 40 μm sampled at 80 μm intervals. This is the equivalent of sampling a one-in-three series of conventional histological sections cut at 40 μm. Using virtual tissue, top and bottom guard zones were not utilized as tissue sectioning artifacts were absent. Imaging through the optical dissector probe height was continuous as a function of the axial resolution of image collection and rendering.

Within each sample section, two regions of interest were defined, a graft core region, which contained the dense distribution of cells and an integration zone, defined as the region outside the graft core extending as far as any cells could be detected. Due to the large differences in cell density, appropriate optical dissector XY dimensions were determined for each region by oversampling/resampling to lower sampling density while maintaining a low coefficient of error^47^. The optical dissector probe was set to 50 μm by 50 μm. Sampling for both regions was performed using a grid step size of 200 μm by 200 μm. The coefficient of error was accepted at or below 0.10.

### Data availability

All data are available from the authors on request.

## Additional information

**How to cite this article:** Doerr, J. *et al*. Whole-brain 3D mapping of human neural transplant innervation. *Nat. Commun.* **8,** 14162 doi: 10.1038/ncomms14162 (2017).

**Publisher's note**: Springer Nature remains neutral with regard to jurisdictional claims in published maps and institutional affiliations.

## Supplementary Material

Supplementary Information Supplementary Figures, Supplementary Tables and Supplementary References

Supplementary Movie 1 Animated 3D reconstruction of a hippocampal lt-NES cell graft (mRFP1, red) showing EGFP+ host input neurons (green) in the medial septal complex, entorhinal cortex, the CA1 sector as well as the hippocampal stratum oriens. Scale bars as indicated.

Supplementary Movie 2 Animated 3D reconstruction of a striatal lt-NES cell graft (mRFP1, red) depicting EGFP+ host cells (green) in cortex as major input source projecting onto the engrafted human neurons. Scale bars as indicated.
