# Supplementary material for: Whole-brain 3D mapping of human neural transplant innervation
Source: Nat Commun. 2017 Jan 19;8:14162. doi: 10.1038/ncomms14162 (PMC5253698; doi:10.1038/ncomms14162)
Supplement: Supplementary Information — Supplementary Figures, Supplementary Tables and Supplementary References [file ncomms14162-s1.pdf]

Supplementary Information

Supplementary Figure 1

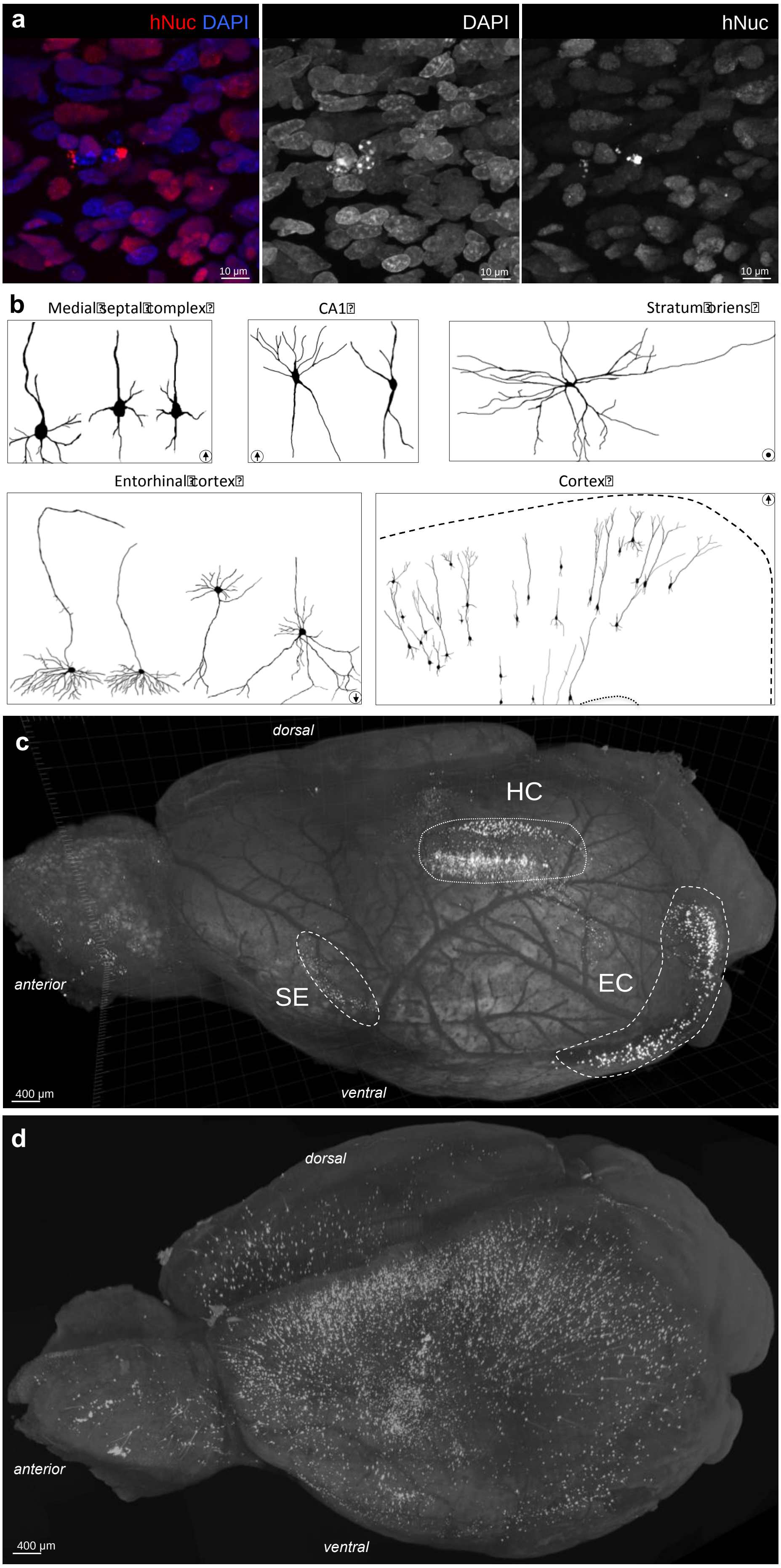

Suppl. Fig. 1. Human graft characterization and transsynaptic labeling of host neurons. (**a**) Graft core (shown for a hippocampal transplant) composed of tightly packed human cells (human nuclei (hNuc; MAB4383; Millipore), red; DAPI, blue). (**b**) Camera lucida drawings of transsynaptically EGFP-labeled neurons in superficial and deep brain regions including medial septal complex, entorhinal cortex, hippocampal CA1 region and stratum oriens (hippocampal graft) as well as neocortex (striatal graft); circled arrows point to the brain surface. (**c-d**) Viral tracing following stereotaxic delivery of a virus cocktail (rAAV (mRFP1/TVA receptor), rAAV (B19 glycoprotein) and RABVΔG-EGFP(EnvA)) into the adult untransplanted hippocampus (**c**) or striatum (**d**) to visualize endogenous innervation of the target area. Analysis was performed 10 days after injection. Scale bars in a, c and d as indicated.

Supplementary Figure 2

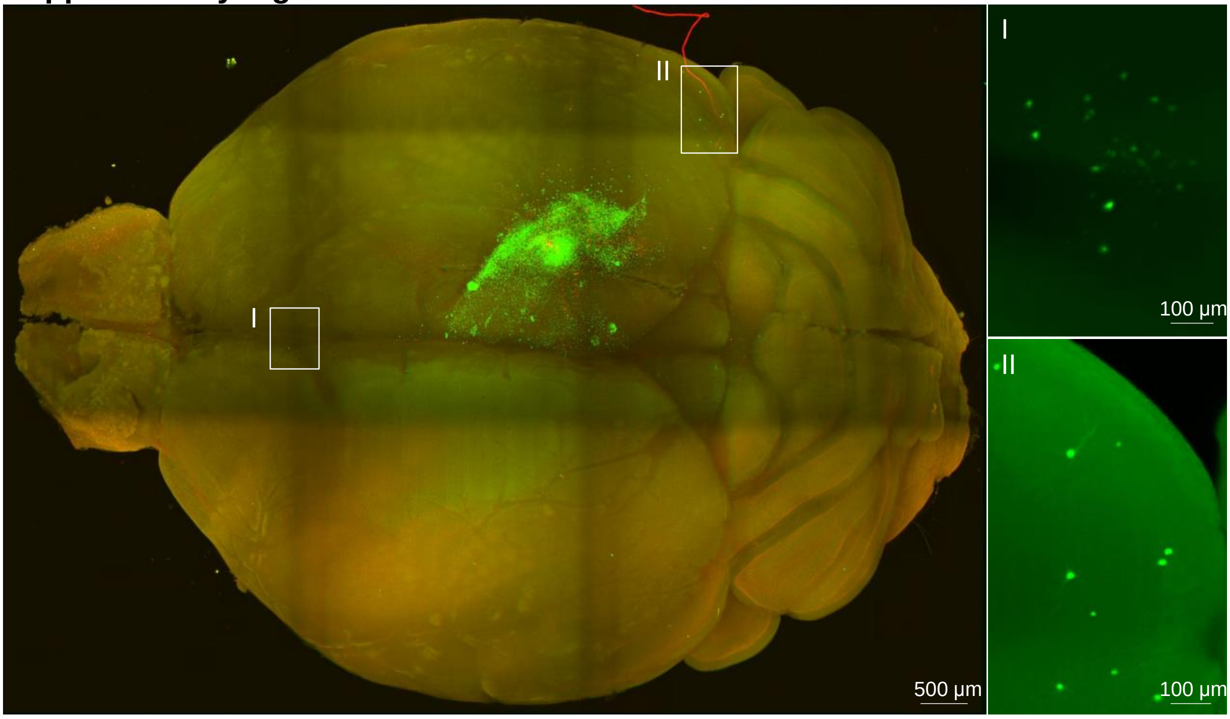

Suppl. Fig. 2. Transsynaptic tracing of a striatal graft of small molecule neural precursor cells (smNPCs) derived from human induced pluripotent stem cells (iPSCs; generated with informed consent of the donor and approved by the Ethics Committee of the University of Bonn Medical Faculty). SmNPCs were generated as previously described [1]. Briefly, induced pluripotent stem cell differentiation was induced by supplementing iPSC growth medium with SB-431542 (10 µM), dorsomorphin (1 µM), purmorphamine (PM, 0.5 µM) and CHIR99021 (CHIR, 3 µM). Evolving smNPCs were propagated in medium containing PM and CHIR. Cells were transduced with the constructs pLVX-SynHTB and pLentiWE-Ef1α-mRFP1, and 100,000 cells suspended in 1 µl of Cytocon Buffer II (Evotec Technologies) were transplanted into the hippocampus of 8-week-old mice. Ten weeks later, the transplant was stereotaxically infected with RABVΔG-EGFP(EnvA). Graft recipients were sacrificed 10 days later, and fixed brain were subjected to tissue clearing and light sheet fluorescence microscopy. EGFP+ labeled donor cells were detected within the hippocampal formation, but also in the medial septal complex (I) and in the entorhinal cortex (II), i.e. characteristic input regions of the hippocampus. Scale bars as indicated.

Supplementary Table 1

Hippocampal grafts

	Animal 1	Animal 2	Animal 3
Transplanted cells	~40,000	~40,000	~60,000
mRFP1-positive cells*	44592	35165	58617
CE**	0.0332	0.0343	0.0455
Absolute number of EGFP-only-positive host cells			
Cortex			
Entorhinal cortex	53	40	47
Occipital lobe	1	0	4
Parieto-temporal lobe	2	5	5
Hippocampal formation	431	450	381
Basal forebrain	0	2	4
Lateral septum	0	4	5
Medial septal complex	47	19	34
Mesencephalic tegmentum	3	0	0
Pons	0	2	0
total	537	522	480

Striatal grafts

	Animal 1	Animal 2	Animal 3
Transplanted cells	~60,000	~60,000	~60,000
mRFP1-positive cells*	56380	75219	67833
CE**	0.0484	0.0417	0.0448
Absolute number of EGFP-only-positive host cells			
Cortex	535	1150	337
Hippocampal formation	0	0	1
Basal forebrain	5	7	4
Medial septum	0	1	0
Striatum	607	312	1045
Globus pallidus	107	53	168
Amygdala	1	1	15
Hypothalamus	2	3	1
Thalamus	36	72	49
Mesencephalic tegmentum	34	47	32
Pons	1	1	0
Total	1328	1647	1652
Regional stratification of EGFP-only-positive cells in cortex			
Primary somatosensory cortex	67	28	2
Primary somatosensory cortex, barrel field	57	129	21
Primary somatosensory cortex, dysgranular zone	0	20	4
Primary somatosensory cortex, forelimb	92	33	40
Primary somatosensory cortex, hindlimb region	15	5	2
Primary somatosensory cortex, upper lip region	53	50	12
Secondary somatosensory cortex	21	32	6
Primary motor cortex	115	215	51
Secondary motor cortex	49	73	30
Insular region (combined)	8	50	17
Lateral orbital cortex	0	240	12
Dorsolateral orbital cortex	5	0	7
Medial orbital cortex	0	4	0
Ventral orbital cortex	0	52	9
Cingulate cortex area 24a	0	18	0
Cingulate cortex area 24b	0	45	9
Cingulate cortex area 24b´	2	0	0
Cingulate cortex area 32	5	12	0
Frontal association cortex	38	115	112
Perirhinal cortex	1	2	0
Piriform cortex	5	12	3
Ventral intermediate enthorinal cortex	0	5	0
Frontal cortex area 3	0	7	0
Secondary auditory cortex, dorsal area	1	0	0
Secondary visual cortex, lateral area	1	3	0
Total	535	1150	337

* cell number determined by stereological analysis ** 2nd estimated coefficient of error (Schmitz-Hof)

Suppl. Table 1. Quantification of grafted human cells and $EGFP^+$ host input neurons. Shown are stereologically determined numbers of engrafted mRFP1-positive cells upon hippocampal or striatal transplantation. CE, coefficient of error, n=3; Stereo Investigator (MBF). EGFP-positive host neurons were quantified using co-registered datasets that allow allocation of fluorescent signals to the MRI-based atlases. Stratification was restricted to the major anatomical subdivisions according to the Allen Mouse Brain Atlas [2].

Supplementary References

1. Reinhardt P*, et al.* Derivation and expansion using only small molecules of human neural progenitors for neurodegenerative disease modeling. *PloS ONE* **8**, e59252 (2013).

2. Lein ES*, et al.* Genome-wide atlas of gene expression in the adult mouse brain. *Nature* **445**, 168-176 (2007).
